# Three-dimensional ultrastructural analyses of anterior pituitary gland expose spatial relationships between endocrine cell secretory granule localization and capillary distribution

**DOI:** 10.1038/srep36019

**Published:** 2016-10-31

**Authors:** Munetake Yoshitomi, Keisuke Ohta, Tomonoshin Kanazawa, Akinobu Togo, Shingo Hirashima, Kei-ichiro Uemura, Satoko Okayama, Motohiro Morioka, Kei-ichiro Nakamura

**Affiliations:** 1 Division of Microscopic and Developmental Anatomy, Department of Anatomy, Kurume University School of Medicine, Kurume, 830-0011, Japan; 2 Department of Neurosurgery, Kurume University School of Medicine, 830-0011, Japan; 3 Electron Microscopic Research Unit, Central Research Unit of Kurume University, Kurume, 830-0011, Japan

## Abstract

Endocrine and endothelial cells of the anterior pituitary gland frequently make close appositions or contacts, and the secretory granules of each endocrine cell tend to accumulate at the perivascular regions, which is generally considered to facilitate secretory functions of these cells. However, three-dimensional relationships between the localization pattern of secretory granules and blood vessels are not fully understood. To define and characterize these spatial relationships, we used scanning electron microscopy (SEM) three-dimensional reconstruction method based on focused ion-beam slicing and scanning electron microscopy (FIB/SEM). Full three-dimensional cellular architectures of the anterior pituitary tissue at ultrastructural resolution revealed that about 70% of endocrine cells were in apposition to the endothelial cells, while almost 30% of endocrine cells were entirely isolated from perivascular space in the tissue. Our three-dimensional analyses also visualized the distribution pattern of secretory granules in individual endocrine cells, showing an accumulation of secretory granules in regions in close apposition to the blood vessels in many cases. However, secretory granules in cells isolated from the perivascular region tended to distribute uniformly in the cytoplasm of these cells. These data suggest that the cellular interactions between the endocrine and endothelial cells promote an uneven cytoplasmic distribution of the secretory granules.

The anterior lobe of pituitary gland (adenohypophysis) is a primary endocrine tissue that consists of five types of endocrine cells, i.e. somatotrophs, lactotrophs, gonadotrophs, corticotrophs, and thyrotrophs, and non-endocrine, folliculostellate cells. It contains abundant capillaries that facilitate the secretory functions of anterior pituitary cells. The hormonal signals from hypothalamus to the pituitary and/or the pituitary to its target organs are thought to diffuse in the intercellular space between endothelial cell and endocrine cells^1^. In this context, the spatial relationship between the capillaries and the endocrine cells plays an important role regulating their secretory function. Previous reports have shown that microvascular density of pituitary tumours is smaller than in normal pituitary gland, and the density varies with adenoma histotypes^2^,^3^,^4^.

Light and electron microscopic studies suggest that a considerable portion of the anterior pituitary gland endocrine cells interacts with the neighbouring capillaries^5^,^6^,^7^,^8^. However, the precise three-dimensional (3D) architecture and spatial relationships between the anterior pituitary gland endocrine cells and capillaries are not well-defined, because the 3D cellular processes are difficult to visualize by immunohistochemical approaches or single-section approaches such as transmission electron microscopy (TEM). Immunohistochemistry using anti-hormone antibodies can be used to visualize the localization of secretory granules (SG) and has frequently been used to visualize the shape of endocrine cells in pituitary gland, but the SGs are not distributed uniformly in the cytoplasms and it is not always possible to visualize all cellular processes using this method. Additionally, some types of endocrine cells are known to extend their cellular process in 3D, but such cellular processes are only observed as a fragment of the cytoplasm in TEM photomicrographs. Therefore the 3D relationships between the cells and their neighboring capillaries are not well defined.

In this study, we analysed the complete 3D architecture and cellular organization of the anterior pituitary tissue in rats, using focused ion-beam scanning electron microscope (FIB/SEM), a novel morphology technique also referred to as “FIB/SEM tomography” that enables visualization of detailed spatial relationships between cells within a tissue^9^,^10^,^11^ FIB/SEM tomography is a scanning electron microscope-based 3D reconstruction method that enables the analysis of large-scale 3D architecture of tissues (ca. 5 × 10^5^ μm^3^) at electron microscopic resolution. It also enables visualization of spatial relationships of many cells in a reconstructed area^9^,^10^,^11^. Using this reconstruction method, we here demonstrated that some endocrine cells do not face the capillaries and are separated from the perivascular space. Additionally, we analysed the distribution patterns of SGs in endocrine cells. As observed by single section analyses, SGs tended to localize and accumulate at secretion sites, such as the juxtavascular regions of the cytoplasm. The mechanism of their specific accumulation at secretion sites is currently unknown. Here, we evaluated the spatial relationship between SG accumulation sites and the apposition between the endocrine and endothelial cells.

## Methods

All experiments were performed in accordance with the National Institutes of Health guidelines for animal research. This study received the approval of the ethics review board of Kurume University Animal Care Centre.

### Specimen preparation

Pituitary glands were obtained from six male Wistar rats(220–250 g). The animals were deeply anesthetized with diethyl ether and peritoneal injection of sodium pentobarbital (50 mg/kg). and left ventricle perfusion with heparin-containing saline (10 U/mL) and fixatives was performed as follows. For immunohistochemistry, the animals (n = 3) were perfused with formaldehyde (4%) in phosphate-buffered saline (PBS) as a fixative, and the pituitary glands were immediately collected and incubated in sucrose (30%)-PBS overnight at 4 °C. The specimens were then embedded in OCT compound (Sakura Finetek, Tokyo, Japan) and frozen. For FIB/SEM tomography, the animals (n = 3) were perfused with formaldehyde (2%) and glutaraldehyde (2.5%) in cacodylate buffer (0.1 M, pH 7.4) as a fixative, and the pituitary glands were then removed and cut into 0.5 mm slices. The specimens were further incubated in the same fixative, 2 h at 4 °C, and washed 3 times with cacodylate buffer (0.1 M, pH 7.4). To enhance membrane contrast, the tissues were post-fixed and *en bloc* stained with heavy metals^12^,^13^,^14^,^15^,^16^,^17^,^18^, as follows. The specimens were immersed in 0.1 M cacodylate-buffered (pH 7.4) 2% osmium tetroxide and 1.5% potassium ferrocyanide solution for 2 h at 4 °C, and then washed five times with distilled water. After that, they were incubated in 1% thiocarbohydrazide solution for 1 h. After five washes with distilled water, they were incubated in aqueous osmium tetroxide solution (2%) for 2 h, and then again washed five times with distilled water. They were immersed in an aqueous solution of 4% uranyl acetate overnight, washed five times with distilled water, and immersed in Walton’s lead aspartate solution for 1 h at 60 °C^19^. After that, the specimens were dehydrated in chilled ethanol series (25%, 50%, 70%, 80%, 90%, and twice in 100%, 10 min each on ice), followed by epoxy resin infiltration (EPON 812, TAAB, England) and polymerization for 72 h at 60 °C. Surfaces of the embedded specimens were exposed using Ultracut E microtome (Reichert-Nissei, Tokyo), the resin blocks trimmed to 1.5 mm^2^ and imaged with SEM (Quanta 3D FEG, FEI, the Netherlands).

### Immunohistochemistry

Cryosections with a thickness of 30 μm were prepared using a HM560 cryomicrotome (Micron, Germany), and released into PBS. The floating sections were then incubated with blocking solution containing 3% normal goat serum and 0.5% Triton X-100 in PBS for light microscopy or 3% normal goat serum in PBS for immune electoron microscopy, and transferred to anti-rabbit ACTH polyclonal antiserumdiluted with blocking solution (1:2000 dilution, Zymed, CA) overnight at 4 °C, followed by rinsing 4 times with PBS. For fluorescence microscopy, addition to the primary antibody, we also reacted with biotin-conjugated *Griffonia simplicifolia 1 lectin* (1:1600 dilution, Vector Labs, CA) for endothelial labeling. The sections were then incubated with Alexa 488-conjugated goat anti-rabbit IgG antibody (1:200 dilution, Invitrogen Life Technologies, CA) as a secondary antibody and Alexa 568 conjugated streptavidin (1:200 dilution, Invitrogen Life Technologies, CA) for 4 h at room temperature. After subsequent rinsing with PBS, the sections were mounted using PermaFluor mounting medium (Thermo Shandon, PA) and observed under a confocal laser scanning microscope (FV1000, Olympus) with acquisition parameters as follows: excitation at 473 nm and 559 nm, x60 oil immersion lens (NA = 1.2), image size = 105 μm × 105 μm. For immunoelectron microscopy, the floating sections were then incubated with biotin-conjugated goat anti rabbit IgG diluted in the blocking buffer (1:200, BA-1000, Vector Labs, CA). After 4 washes with PBS, the sections were reacted with ABC complex solution (Nacalai Tesque, Kyoto, Japan) and then the DAB reaction was performed following the manufacturer’s instructions. The specimens were dehydrated with a gradient series of acetone and embedded in epoxy resin (EPON812, TAAB, England). Ultrathin sections of the specimens were stained with saturated uranyl acetate solution and examined using transmission electron microscopy (H-7000, Hitachi).

### FIB/SEM tomography

Serial stacked images for 3D reconstruction were obtained using an FIB/SEM apparatus (FEI Quanta 3D FEG, Netherlands) as described previously. To obtain high-contrast images, we applied sample bias voltage, also referred to as retarding, during the image acquisition for part of the reconstruction. The specimens were set on a originally manufactured pre-tilted holder that prevented sample bias-derived severe astigmatism during the image acquisition^17^. Specimen milling and imaging were performed as follows. The gallium ion beam for specimen milling was set at 30 kV, with a current of 5 nA. The milling pitch was set to 50 nm/step (BSE) or 100 nm/step (SE). Images were acquired using either back-scattered electrons (BSE) with sample bias or secondary electrons (SE) without sample bias, under the following conditions. All image sizes were set at 2048 × 1766 pixels with a pixel resolution of 50 nm (99.5 μm × 85.9 μm). Conditions for BSE images were detector = VCD (FEI), acceleration voltage = 5 keV of landing energy with 1 kV of sample bias, beam current = 13.6 pA, and dwell time = 30 μs/pixel. Conditions for SE images were detection = Everhart-Thornley (FEI), acceleration voltage = 2.5 keV (without sample bias), beam current = 50 pA, and dwell time = 10 μm/pixel. The milling and imaging were repeated 500 or 600 times.

### 3D reconstruction and segmentation

The shapes of cells, SGs, and blood vessels were segmented and annotated from the 3D reconstruction volume data using Avizo 6.5 software (FEI Visualization Sciences Group, Bordeaux, France). After the image alignment, lumens of blood vessels were segmented automatically with a threshold method, while cellular shapes were selected manually. Only cells entirely within the reconstructed volume were subjected to 3D analysis. SGs were further segmented from the cytoplasm as an electron dense region using a cell-by-cell threshold method.

### Cell classification

Anterior pituitary cells were classified into five types according to their ultrastructural characteristics, as previously reported^20^: type 1, polygonal cells with very small SGs (smaller than 100 nm); type 2, extended cells with long irregular granules (400–700 nm); type 3, large irregularly-shaped cells with small SGs (150–200 nm) predominantly in the peripheral region of the cytoplasm; type 4, cells of intermediate size, with round dense granules (about 350 nm); type 5, long cells with SGs (200 nm or 500 nm in diameter). Different cell types secrete different hormones, as follows: thyroid-stimulating hormone-producing thyrotrophs (TSH, type 1), luteotropic hormone-producing lactotrophs (LTH, type 2), adrenocorticotropic hormone-producing corticotrophs (ACTH, type 3), somatotropin hormone-producing somatotrophs (STH, type 4), gonadotropin hormone-producing gonadotrophs (GTH, type 5)^20^,^21^,^22^.

### Statistical analysis of granule distribution pattern

We used type 4 cells (somatotroph) for statistical analyses of the unevenness of SG distribution. Measurable cells were selected from the reconstructions in both “contact” (n = 20) and “isolated” (n = 12) categories. First, we calculated the centre of gravity (CG) of the area of the whole cell using Avizo software. Next, we extracted the SG area in the cell based on its high electron density using simple thresholding and calculated the CG of the extracted SG area. Then, the distance between two points, i.e., between the CG of the whole cell and the CG of the SG area, was calculated in each cell. The obtained values were analysed using JMP version 11 (SAS Institute Inc. Cary, NC, USA). Comparisons between groups were performed using the Wilcoxon rank-sum test. Differences were considered significant at *p* < 0.05.

## Results

### FIB/SEM tomography allows 3D reconstruction of the anterior pituitary gland

**Figure 1:**
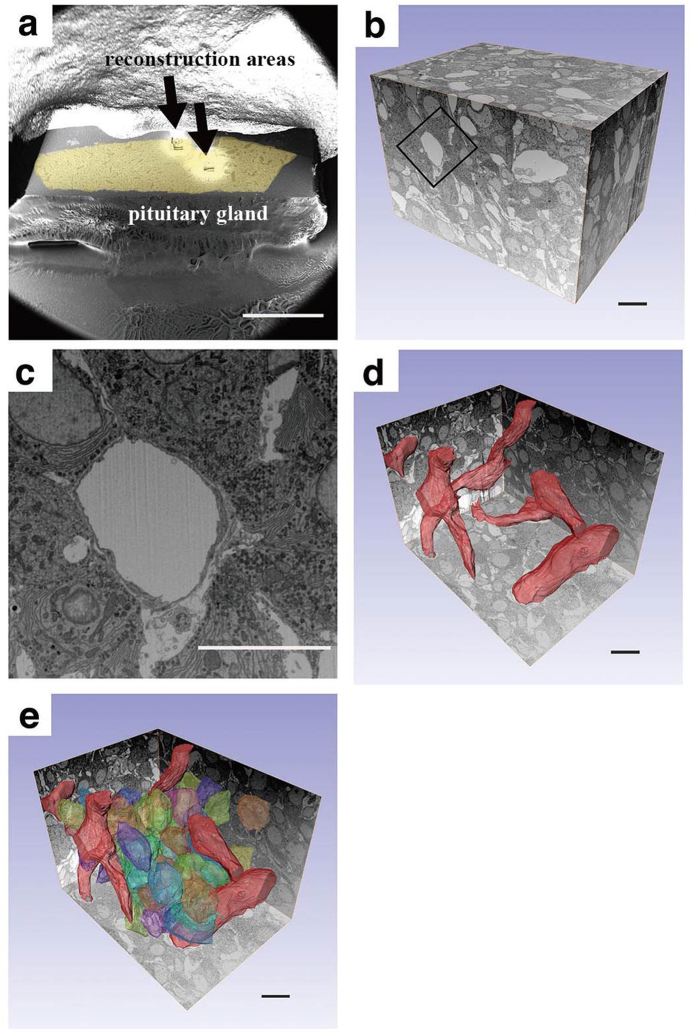
Three-dimensional FIB/SEM tomography reconstruction of the anterior pituitary gland. (**a**) Low-magnification SEM micrograph showing the entire resin-embedded specimen after data acquisition. Small pimples (arrows) comprise the reconstructed area. (**b**) Stack of serial images. (**c**) Higher magnification view of the area enclosed by a rectangle in (**b**). (**d**,**e**) 3D-rendered views of blood vessels (**d**) and endocrine cells together with the blood vessels (**e**). Scale bars, (**a**) 1 mm; (**b**–**e**) 10 μm.

We used FIB/SEM tomography to examine rat anterior pituitary glands. Regions for 3D reconstruction were randomly selected from the whole resin-embedded glands (Fig. 1a coloured area), and 500 to 750 serial images were acquired. The final reconstruction size was approximately 100 × 100 × 50 μm^3^ to 100 × 100 × 75 μm^3^ (Fig. 1b). Each block face image had sufficient resolution to characterize SGs (Fig. 1c) and also distribution pattern of the SGs, but the reconstruction was not sufficient to analyse the shape of each SG, especially in the case of Type 1 cells, because the size of the SG was equivalent to almost 2 pixels of the image. Although the depth resolution of the reconstructions was lower than that of the lateral resolution of the individual block face image, it was sufficient to obtain detailed 3D profiles of vessel networks (Fig. 1d) and endocrine cells (Fig. 1e). Three segmentations showed that each reconstruction contained approximately 100 cells.

### FIB/SEM tomography facilitates endocrine cell typing

**Figure 2:**
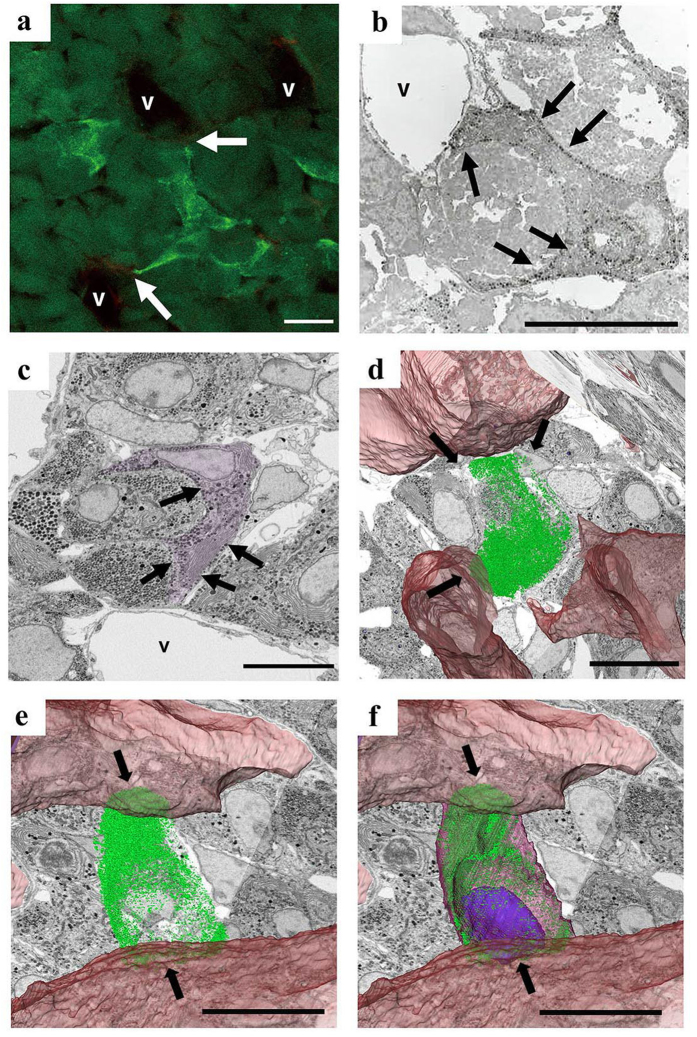
Light microscope and ultrastructural characterization of type 3 endocrinal cells embedded in pituitary gland tissue. (**a**) Immunohistochemical micrograph of ACTH cells extending cellular processes (arrows) toward the blood vessels (V). (**b**) Immunohistochemical transmission electron micrograph of ACTH cells showing cellular processes (arrows) extended toward the blood vessels. (**c**) Single slice images from a stack, acquired using FIB/SEM. Type 3 cell (which may be interpreted as an ACTH cell) is false-coloured purple. (**d**,**e**) Different views of 3D distribution of secretory granules (green) in the cell from panel (**c**). (**f**) View from (**e**) showing the entire cellular shape with nucleus (blue). Although this cell shows a process-like cytoplasmic extension toward the vessel in 2D view (arrows in **c**), 3D reconstruction demonstrated that this extension is part of sheet-like cellular projections attached to pericapillary space (arrows in **d–f**). Bar scale: 10 µm in all panels.

In this study, we analysed three pituitary glands and counted 96, 72, and 98 endocrine cells in reconstructions 1, 2, and 3, respectively. The milling pitch and final reconstruction size of reconstructions are summarized in supplemental Table 1. These cells were classified into five types according to the shape and distribution pattern of SGs, as mentioned in the Method section (Table 1). For example, cells with small SGs (150–200 nm) localized in the peripheral cytoplasmic region, which were classified as type 3, were considered to be consistent with ACTH cells^20^ (Fig. 2a). We also verified that cells having such a distribution of SGs coincided with ACTH cells by fluorescence microscopy and immunoelectron microscopic detection of ACTH (Fig. 2b). Such a unique SG distribution pattern was also observed in the FIB/SEM reconstruction volume (Fig. 2c), and we classified the cells as Type 3 cells in this study. The 3D distribution pattern of SGs in the cell is shown in Fig. 2d–f. The distribution pattern in Fig. 2d and e indicated a slender and cytoplasmic process-like shape (Fig. 2d,e, arrows), which was similar to the pattern in immunohistochemistry (Fig. 2a). However there were cells that difficult to classify into the 5 types based only on their SG characteristics, and we labelled them as “others” in Table 1.

### Spatial relationships between endocrine and endothelial cells reveal the existence of apposed and isolated endocrine cells

**Figure 3:**
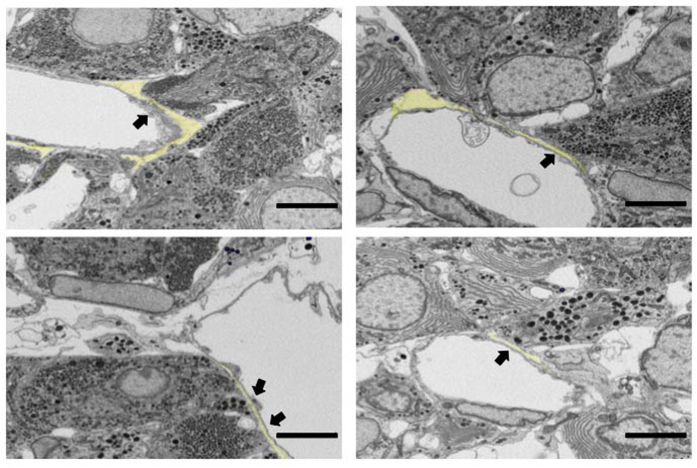
Apposition between endothelial and endocrine cells. Block-face images of rat anterior pituitary gland obtained using scanning electron microscopy. Endocrine cells are apposed only around the endothelial cells of the capillaries. Small gaps between endothelial and endocrine cells are frequently observed (coloured area), and no apparent physical junction structures are observed. Secretory granules in the endocrine cells tend to accumulate in the area apposed to endothelial cells (arrows). Bar scale: 5 µm in all panels.

**Figure 4:**
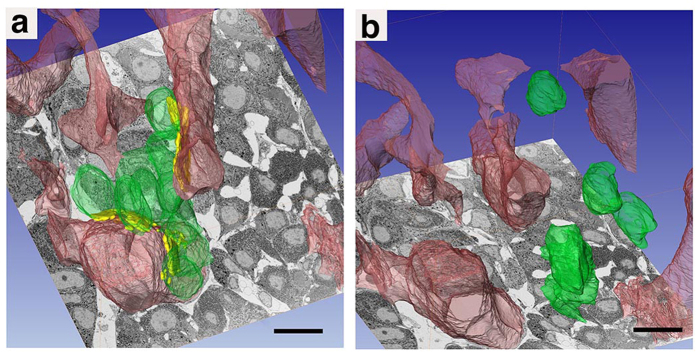
FIB/SEM 3D characterization of “apposed” and “isolated” endocrine cells. FIB/SEM 3D reconstruction demonstrated that about 70% of the anterior pituitary endocrine cells were located in the proximity of blood vessels, with the remaining 30% not involved in any apparent physical contact with the vessel wall and isolated from the perivascural space (Table 1). (**a**) “Apposed” (green) and endothelial (red) cells are shown, with apposition regions indicated in yellow. (**b**) “Isolated” (green) and endothelial (red) cells in another tissue region. Bar scale: 10 µm in all panels.

**Figure 5:**
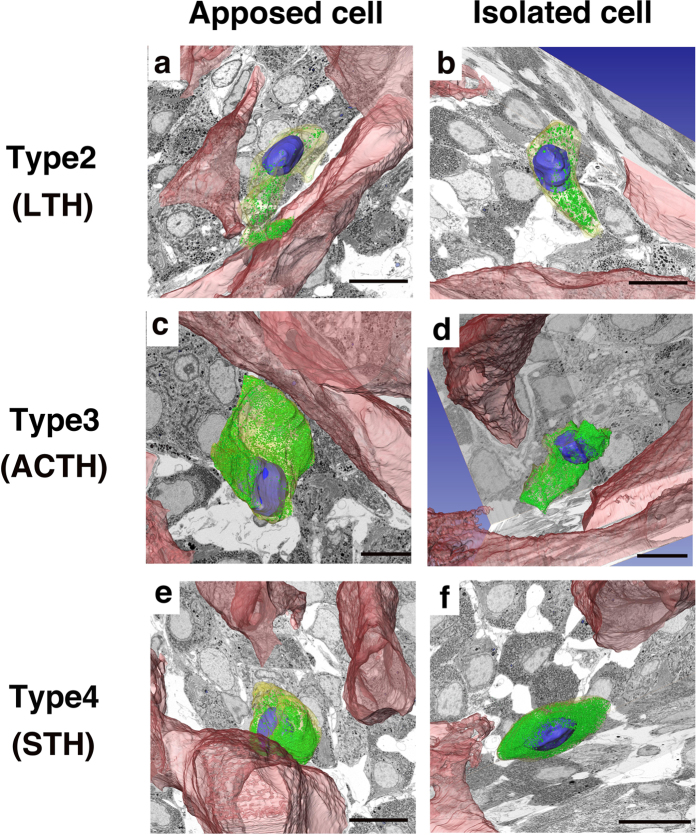
Secretory granule (SG) distributions in the anterior pituitary endocrine cells. 3D distributions of SGs were visualized by FIB/SEM 3D reconstruction data. Representative distribution patterns of SGs (green) in types 2, 3, and 4 endocrine cells were compared in apposed cells that contacted the endothelial cells, and isolated cells that did not physically interact with endothelial cells. The SGs in apposed cells are tended to accumulate in the periphery of the cell cytoplasm, especially around the perivascular area (**a,c,e**), but the SGs in the isolated cells were observed all around the cytoplasm (**b,d,f**). Bar scale: 10 µm in all panels.

We analysed the spatial relationships between endothelial cells and each endocrine cell type. Some endocrine cells that localized around the blood vessels faced the capillary walls. We observed a small gap (ca. 600 nm) between these cells and the endothelial cells and no specialized junction structures (Fig. 3, coloured area). Furthermore, SGs in these cells tended to accumulate in regions facing the endothelial cell (Fig. 3, arrows). In this paper, we name the regions where endocrine cells directly face endothelial cells “apposition sites” (Fig. 4a), and we term cells with more than one apposition site “apposed.” When no apposition sites were seen between endocrine cells and the capillary walls (Fig. 4b), we considered the endocrine cells “isolated” (Table 1). Since the apposition site sizes vary depending on the relative positioning of cells, we only counted apposition sites where the long axes exceeded 500 nm. Approximately 31% of all endocrine cells detected by us were “isolated” (Table 1) and such isolated cells were observed for all endocrine cell types (Table 1, Figs 4 and 5).

### Secretory granule distribution in apposed cells is different from that in isolated cells

**Figure 6:**
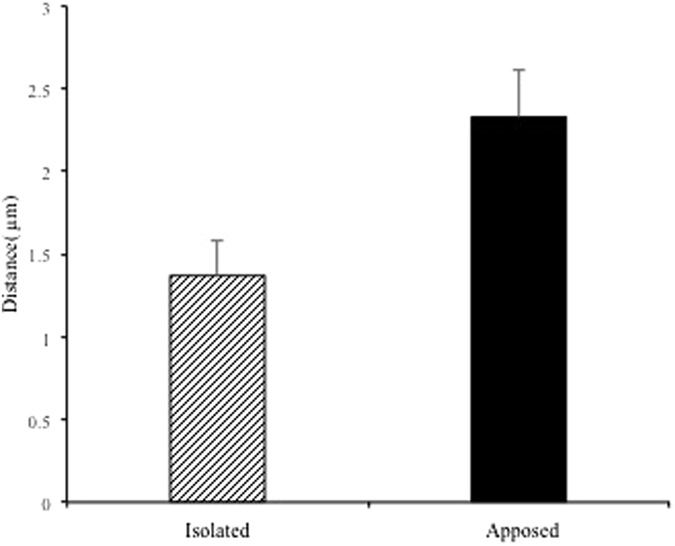
Analysis of secretory granule (SG) distribution in type 3 “apposed” and “isolated” endocrine cells. Distances between the centres of gravity for the cells and for the SGs in the cells were compared between “apposed (n = 20)” and “isolated (n = 12)” in type 4 endocrine cells. Statistical analyses were performed and revealed a significant difference between these two sorts of cells (Wilcoxon signed-rank test, *p* < 0.05). The data are presented as means ± standard error.

3D reconstruction revealed that SGs in cells facing the blood vessels tended to localize in a peripheral cytoplasmic region, close to the apposition site (Fig. 5, left panels). Type 2 cells (LTH) frequently had long cellular processes and many of their SGs accumulated at the perivascular space (Fig. 5a). Type 3 cells (ACTH) usually had a widespread sheet-like cytoplasm (also see Fig. 2f), and SGs naturally localized along the juxta-membrane area (Fig. 2b,c), whereas some SGs accumulated around the perivascular area (Fig. 2f and Fig. 5c). Type 4 cells (STH) had a round-shaped cytoplasm (Fig. 5e), and their SGs accumulated around the perivascular region. In contrast, SGs in cells isolated from the perivascular space appeared to be distributed uniformly in the cytoplasm (Fig. 5, right panels). We obtained a sufficient number of type 3 cells in both the “apposed” and “isolated” state to perform statistical analyses. We measured the CG of each cell and also the CG of the area occupied by SGs within the cell, as described in the Methods section. We then calculated the distance between these points cell by cell. If the SGs were distributed uniformly in the cell, both points were in almost the same position, and the distance between the two CGs became small. In contrast, when SGs were localized in the peripheral terminal of the process, the distance between the CGs tended to become large. In our study, we estimated the distance between CGs in type 4 cells of the isolated group and apposed group, and found that the distance between CGs in the apposed group was significantly larger than that in the isolated group, which suggests that the SGs in the apposed group are more unevenly distributed than those in the isolated group (Fig. 6).

## Discussion

In this study, we used FIB/SEM tomography to visualize the cyto-architecture of the anterior pituitary gland with ultrastructural resolution, focusing on the spatial relationships between the endocrine and endothelial cells. We also evaluated the 3D distribution patterns of SGs in each hormone-secreting cell type since the 3D reconstruction data afford sufficient resolution to visualize not only the cellular organization of tissue, but also intracellular spatial localization of SGs.

Each reconstructed region (volume) contained about 100 cells and our data provided enough information to analyse their structural properties. Each acquired image confirmed the classical TEM observations (data not shown), and we were able to classify the endocrine cells into five types, according to their morphological characteristics^20^,^21^,^22^. Indeed, the same endocrine cell types extracted from the reconstructed images had a relatively similar 3D appearance (e.g. Figs 2 and 5), which reflected their previously reported cellular characteristics. For example, a typical adult ACTH cell, verified immunohistochemically, frequently has long cytoplasmic processes extending toward the capillaries^23^, and this characteristic was seen in our 3D reconstructions when we selectively visualized SGs, as shown in Fig. 2. However, the true 3D structure of a cell is somewhat different from its immunohistochemical characteristics. Large proportion of type 3 cells frequently possessed a sheet-like cytoplasmic extension whose tip, not a fine cellular process, resided in the pericapillary space. Such 3D characteristics of the cell were difficult to glean from only immunohistochemical observation, even when we reconstructed the structure of the cell in 3D using confocal light microscopy, because this technique visualized only the distribution pattern of the hormone in the cell. In contrast, our 3D model easily explained the discrepancy between the immunohistochemical appearance of ACTH cells and the true 3D structure of the cell.

Our imaging experiments clearly visualized the spatial relationship between endocrine cells and vasculature. We demonstrated that about 30% of endocrine cells in the parenchyma of the anterior pituitary gland do not physically interact with endothelial cells. This finding was unexpected since it is widely accepted that a large proportion of the anterior pituitary gland endocrine cells physically interacts with capillary blood vessels^24^. Developmental studies have revealed that differentiation of the endocrine cells is closely linked to the blood vessels infiltrating the pouch of Rathke^25^,^26^,^27^. Confocal microscopy and multiphoton microscopy studies of topological relationships between endocrine cells and blood vessels reported an evident interaction, interpreted as a framework that facilitates the secretion of hormones. This interaction was confirmed by conventional TEM experiments that evidenced a direct contact between endocrine and endothelial cells^20^. Our data revealed that 70% of endocrine cells were closely apposed to the perivascular space.

Discovery of endocrine cells isolated from the perivascular space enabled us to attempt to correlate the SG distribution with different cellular interactions (apposed vs. isolated). In our observation, the SGs tended to distribute near the terminal of cellular processes located at perivascular spaces, even in 3D, and this agreed with previous reports^24^. In contrast, we observed for the first time that SGs in cells without contact to capillaries appeared to be localized more evenly in the cytoplasm. Therefore, we tried to estimate the difference in SG localization in the cells by using the difference in the absolute position of CGs between the whole cellular area and the SG area in the cell. Our statistical analysis revealed that in type 4 cells (somatotrophs), the cytoplasmic SG accumulation was uneven, especially in the vicinity of the endothelial cells. Previous ultrastructural studies indicated that SGs within anterior pituitary cells, such as ACTH or LTH cells, tended to accumulate in the juxtavascular cytoplasm^28^. Similar accumulation of SGs has been also observed in other endocrine cells, e.g. pancreatic islet cells and basal granulated cells of the intestinal endothelium. These accumulations are thought to be facilitated by a specific intracellular transport system, the regulated secretory pathway (RSP), which carries the SGs to the cytoplasmic area close to the blood vessels and facilitates efficient hormone secretion^29^. RSP comprises a multiple step post-Golgi vesicular transport system that regulates exocytosis and it is specifically observed in endocrine cells^29^,^30^. In this study, we showed a dispersed cytoplasmic distribution of SGs within the “isolated” endocrine cells compared to the “apposed” cells. This suggested that the physical interactions between the endocrine and endothelial cells might induce specific RSP polarity, from the site of biogenesis to the secretion site, close to the vessels. Our reconstruction had sufficient resolution to visualize physical junctions such as desmosomes (data not shown), but we did not observe obvious junctional structures between endothelial and endocrine cells in our 3D analysis. It is possible that focal adhesions were present, but we could not evaluate the formation of focal adhesions in this study because of the resolution. Our findings raise the possibility that some cytokines or paracrine factors, in addition to focal adhesions, may induce cellular RSP polarity, although the molecular mechanism is not known. In the nervous system, humoral factors such as netrins, slits, and semaphorins, induce cellular polarity during neural guidance^31^,^32^. These humoral factors induce the extension of axons to create the majestic neuronal networks. Similar humoral mechanisms might perhaps be involved in inducing cellular elongation of the endocrine cells in the direction of vessel walls.

What is the significance of “isolated “endocrine cells that do not contact the blood vessel walls? It is possible that these cells simply remain within the parenchyma as a result of the developmental process but have no function. During the early development of the anterior pituitary gland, most cells are thought to differentiate into mature endocrine cells because of the infiltration of the portal vein. Consequently, they are localized around the vessels, following which these endocrine cells proliferate and begin to extend their cytoplasmic processes toward the vessels^6^. Some of these processes possibly retract during development and are isolated from the pericapirally space. However, most of “isolated” cells observed here contained abundant SGs within their cytoplasm. We therefore speculate that these cells may contribute to the secretory function of the anterior pituitary gland. Experiments with dextran bead injections into the pituitary gland suggested that hormones might be transported into the bloodstream through intercellular spaces via diffusion^33^. This implies that the “isolated” cells may contribute to hormone secretion and the secreted hormones would reach the vessel by diffusion, but the diffusion time required for transportation along the intercellular channel from isolated cells to the bloodstream may be longer than that required a cell adjacent to the vessel wall. These morphological differences would result in a different hormone release mode, e.g. sharp and short secretion in apposed cells and slower and smoother secretion in isolated cells.

Our 3D analysis demonstrated novel structural characteristics of the anterior pituitary gland. Cyto-architectural analyses alone are insufficient for fully characterising the hormone secretion mechanisms, but the increased understanding of the actual tissue architecture that they provide will pave the way for characterising the novel, topological, anterior pituitary regulation of systemic hormone secretion.

## Additional Information

**How to cite this article**: Yoshitomi, M. *et al*. Three-dimensional ultrastructural analyses of anterior pituitary gland expose spatial relationships between endocrine cell secretory granule localization and capillary distribution. *Sci. Rep.* **6**, 36019; doi: 10.1038/srep36019 (2016).

**Publisher’s note:** Springer Nature remains neutral with regard to jurisdictional claims in published maps and institutional affiliations.

## Supplementary Material

Supplementary Information

## Figures and Tables

**Table 1:** Classification of endocrine cells in the 3D reconstruction volume according to their ultrastructural properties.

Cell classification^1^	Apposed cell^2^	Isolated cell^3^	Total
Type1 (TSH)	26	3	29
Type2 (LTH)	17	2	19
Type3 (ACTH)	13	7	20
Type4 (STH)	51	28	79
Type5 (GTH)	43	24	67
Others	36	11	47
Total	181	83 (31.4%)	264

^1^Cell types: type 1 (thyroid-stimulating hormone-producing, TSH), type 2 (luteotropic hormone-producing, LTH), type 3 (adrenocorticotropic hormone-producing, ACTH), type 4 (somatotropin hormone-producing, STH), type 5 (gonadotropin hormone-producing, GTH).

^2^Cells that physically interact with endothelial cells.

^3^Cells that do not physically interact with endothelial cells.
