# Supplementary material for: Three-dimensional ultrastructural analyses of anterior pituitary gland expose spatial relationships between endocrine cell secretory granule localization and capillary distribution
Source: Sci Rep. 2016 Oct 31;6:36019. doi: 10.1038/srep36019 (PMC5086841; doi:10.1038/srep36019)
Supplement: Supplementary Information [file srep36019-s1.pdf]

Three-dimensional ultrastructural analyses of anterior pituitary gland expose spatial relationships between endocrine cell secretory granule localization and capillary distribution

Munetake Yoshitomi[1,2*], Keisuke Ohta[1,3], Tomonoshin Kanazawa[1], Akinobu Togo[3], Shingo Hirashima[1], Kei-ichiro Uemura[1], Satoko Okayama[1], Motohiro Morioka[2], Kei-ichiro Nakamura[1]

[1]Division of Microscopic and Developmental Anatomy, Department of Anatomy, Kurume University School of Medicine, Kurume, 830-0011, Japan.
[2]Department of Neurosurgery, Kurume University School of Medicine, 830-0011, Japan.
[3]Electron Microscopic Research Unit, Central Research Unit of Kurume University, Kurume, 830-0011, Japan.

***Corresponding author: munetake06@gmail.com**

supplement table

	slice pitch	reconstructed volume (μm)	cell number
Rec1	50nm	98.0 x 85.7 x 52.1	96
Rec2	100nm	92.9 x 64.2 x 68.4	72
Rec3	100nm	93.0 x 64.2 x 68.4	98
